# Extracellular presentation of syntaxin4 as a potential trigger for region-specific gastrulation

**DOI:** 10.1247/csf.25073

**Published:** 2025-10-29

**Authors:** Sae Nozaki, Taisei Mihara, Yohei Hirai

**Affiliations:** 1 Department of Biomedical Sciences, School of Biological and Environmental Sciences, Kwansei Gakuin University, 1, Gakuen-Uegahara, Sanda, 669-1330, Japan

**Keywords:** gastrulation, FAK, P-cadherin, Rho/ROCK, membrane flip

## Abstract

During early embryogenesis, gastrulation occurs within a specific region of the pluripotent epiblast, where cells undergo significant changes in their context. The induction of these cellular transformations in particular cell populations suggests the involvement of non-diffusible factors that activate signaling pathways in a spatiotemporal manner. Syntaxin4 (Stx4), a type IV membrane protein that functions as an intravesicular fusion mediator, often translocates across membranes to perform a latent extracellular role in locally regulating cellular behaviors. Through the culture of mouse embryonic egg cylinders isolated from E6.0 embryos and embryonic stem cells (ESCs), we demonstrate that the membrane translocation of Stx4 may play a crucial role in this early stage of development. Using membrane-impermeable antagonistic peptides against extracellular Stx4, along with several small-molecule inhibitors and activators, we found that cells with extracellular Stx4 deactivate focal adhesion kinase (FAK), which then impacts AKT/PI3K signaling and results in increased expression of P-cadherin, ultimately inducing the expression of the gastrulation marker brachyury. Activation of this signaling pathway also triggers Rho/ROCK signaling in ESCs, leading to morphological changes. These findings offer important insights into gastrulation by shedding light on the molecular mechanisms that initiate the spatiotemporal changes in the uniform pluripotent cell sheet.

## Graphical Abstract

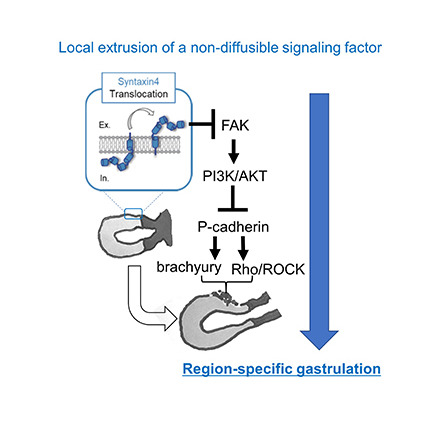

## Introduction

Gastrulation is a vital event in early embryogenesis, during which the epiblast undergoes significant morphological changes and loses its stem cell properties to form the three primary germ layers: ectoderm, mesoderm, and endoderm. This process is driven by complex molecular mechanisms involving various signaling pathways and gene regulatory networks (Bardot and Hadjantonakis, 2020; Molè *et al.*, 2020). During gastrulation, a specific region of the pluripotent epiblast experiences morphological transformation and differentiation, marking the start of embryonic development. The dynamic cell behaviors observed during this stage have long been a focus of scientific research, and several underlying mechanisms have been identified. For example, diffusible factors such as Wnt, Bone Morphogenetic Protein (BMP), and Nodal are known to establish the body axis and coordinate cellular movements (Beddington and Robertson, 1999; Morgani and Hadjantonakis, 2020; Siggia and Warmflash, 2018). Transcription factors, including Snail, Slug, and Twist, regulate gene expression programs that control cell morphology, migration, and differentiation, thereby specifying lineage (Carver *et al.*, 2001; Moly *et al.*, 2016; Wong *et al.*, 2014).

Although gastrulation is a highly coordinated process involving complex crosstalk between molecular signals, gene regulation, and cellular dynamics, it begins in a spatially restricted area of the initially uniform epiblast, and the exact nature of the initiating cues remains only partially understood (Mikawa *et al.*, 2004).

Before the start of gastrulation, some changes are observed in restricted cells, including loss of cell polarity (Weiser and Kimelman, 2012), changes in cytoskeletal dynamics (Roh-Johnson *et al.*, 2012), and weakening of intercellular adhesion systems (Basilicata *et al.*, 2016; Collins and Nelson, 2015), partly mediated by the downregulation of E-cadherin and the upregulation of P-cadherin (Acloque *et al.*, 2017; Moly *et al.*, 2016). Given that gastrulation occurs within a specific cell population at a restricted time point during early embryogenesis, we hypothesize that non-diffusible proteins with spatiotemporally restricted functions may play a crucial role in initiating this process. As candidate proteins, we focused on type IV membrane proteins, plasmalemmal syntaxins, which typically function as t-SNARE components on the cytoplasmic surface of membranes but are transiently translocated to the cell surface, where they activate latent signals and locally regulate cell behavior. While these distinctive properties were initially identified for syntaxin2 (epimorphin) (Hirai *et al.*, 2007; Radisky *et al.*, 2009), this protein contains a unique site for proteolytic cleavage within its C-terminal, membrane-proximal domain, resulting in partial secretion (Hirai *et al.*, 2007). In contrast, Syntaxin4 (Stx4) lacks such a site, and unlike epimorphin, most of the extruded form remains anchored at the cell surface, exerting pronounced effects only on the behavior of Stx4-extruding cells and their neighbors (Hirai *et al.*, 2007; Kadono *et al.*, 2012). For example, in mammary epithelial cells, hormonal stimulation facilitates the translocation of this protein across the membrane in particular cell populations, which locally induces morphological changes (Hirose and Hirai, 2021; Shirai *et al.*, 2017). In skin keratinocytes, calcium influx leads to the presentation of its cell surface in stratified keratinocytes, which promotes epidermal differentiation (Kadono *et al.*, 2015). In mouse embryonic stem cells (mESCs), an extracellular form of Stx4 becomes locally detectable upon spontaneous differentiation, and extracellular Stx4 (ExStx4) has been shown to cause morphological changes and a loss of stemness (Hagiwara-Chatani *et al.*, 2017; Matsuguchi and Hirai, 2023). Notably, ExStx4 strongly upregulates the classic gastrulation marker Brachyury and the gastrulation onset marker P-cadherin (Hagiwara-Chatani *et al.*, 2017), and mice lacking Stx4 experience embryonic lethality soon after the gastrulation period (Yang *et al.*, 2001; Yoo *et al.*, 2015). Additionally, transcriptome analysis of doxycycline-inducible ExStx4 in mES cells shows that ExStx4 also alters the expression of several other gastrulation-related genes, including decreasing stemness markers *Nanog* and *Zscan*, increasing the actin regulator *CFL1* (cofilin 1 gene), and raising the levels of key gastrulation-supporting genes such as *FGF8* (fibroblast growth factor 8 gene) (Hagiwara-Chatani *et al.*, 2017).

Recently, we examined the molecular components involved in ExStx4-induced signaling using NCCIT cells (Nozaki and Hirai, 2023). This highly stable human embryonic carcinoma cell line can differentiate into all three germ layers. Although NCCIT cells usually do not display Stx4 on their surface under standard culture conditions, artificial expression of ExStx4 triggered responses similar to those in ESCs. These NCCIT responses were driven by Rho/ROCK activation and simultaneous inactivation of PI3K. Additionally, we found that this regulation occurred downstream of FAK inactivation and P-cadherin upregulation, both of which were induced by ExStx4 in these cells (summarized in Supplementary Fig. S1).

In this study, we explored the role of these molecular elements in early development using organ culture of embryonic egg cylinders and cell culture of fully functional mESCs. We examined the expression of *Nanog* as a stemness marker, *CDH3* (known as P-cadherin gene) as an indicator of the onset of gastrulation, and *T* (known as Brachyury gene) as a marker of gastrulation. Our observations revealed similar mechanisms underlying the significant cellular dynamics induced by ExStx4, with some notable differences. These results significantly improve our understanding of the molecular processes involved in mammalian gastrulation, especially in the limited area of the pluripotent epiblast.

## Materials and Methods

### Cells

A human embryonic carcinoma cell line NCCIT (NCCIT-A3, JCRB Cell Bank) and their derivatives, previously isolated in our study (Nozaki and Hirai, 2023), were cultured in RPMI medium (FujiFilm-Wako, Osaka, Japan) supplemented with 10% fetal bovine serum (FBS), 50 U/ml penicillin, and 50 μg/ml streptomycin (Meiji Seika Pharma, Tokyo, Japan). mESCs (E14-Tg2A) and those carrying a dox-inducible expression cassette of Stx4 containing an N-terminal fusion of an exogenous signal peptide and T7-tag (mESCs-ExStx4) or P-cadherin (mESCs-P-cad) (Hagiwara-Chatani *et al.*, 2017; Takeda *et al.*, 2023) were maintained on 0.1% gelatin-coated dishes using Glasgow’s Minimum Essential Medium (FujiFilm-Wako) supplemented with 10% fetal bovine serum (FBS), 1 mM glutamine (Thermo Fisher Scientific, Waltham, MA, USA), 1 mM sodium pyruvate (FujiFilm-Wako), 0.1 mM non-essential amino acids (Sigma Aldrich, St. Louis, MO, USA), and 0.1 mM β-mercaptoethanol (FujiFilm-Wako). To maintain the stemness of the cells, the culture medium also contained leukemia inhibitory factor (LIF) and a combination of 1 mM PD0325901 and 3 mM CHIR99021 (known as 2i) (Thermo Fisher Scientific). For transgene expression in derivatives of NCCIT cells and mESCs, 5 μg/ml doxycycline (dox) was added to the medium, and the cells were cultured for three days. To generate mESCs-ExStx4 with genetic ablation of P-cadherin, mESCs-ExStx4 were transfected with the P-cadherin-knockout (KO) plasmid using the CUY21Pro-Vitro electroporator (Nepa-gene, Ichikawa, Japan). They were then treated with 5 μg/ml puromycin (Invitrogen-Thermo Fisher, Waltham, MA, USA) for three days and subsequently cloned. Each isolated clone was cultured and screened for frameshift mutations in the *CDH3* gene. For constructing the P-cadherin-KO plasmid, synthetic cDNAs for two guide RNAs (gRNA) were designed around the translation start codon: 5'-CACCgttcgtccccgagaatggcaa-3' annealed with 5'-AAACttgccattctcggggacgaac-3' (for gRNA1); and 5'-CACCgaccattagcgtcatatccag-3' annealed with 5'-AAACctggatatgacgctaatggtc-3' (for gRNA2). One of the annealed cDNAs was inserted into the Bbs I site in pSpCas9 (BB)-2A-Puro (Addgene, Watertown, MA, USA). Clones isolated using gRNA2 exhibited slower growth compared to parental mESCs-ExStx4; therefore, clones isolated using gRNA1 were used.

### Small molecule inhibitors and activators

Selective inhibitors used in this study included those targeting ROCK (Y27632), myosin light chain kinase (MLCK) (ML-7), Rac1 (NSC23766, FujiFilm-Wako), PI3K/AKT (LY294002), and FAK (VS4718, ChemieTek, Indianapolis, IN, USA). The FAK activator ZINC40099027 was obtained from AOBIOUS (Gloucester, MA, USA). Y27632, ML-7, and NSC23766 were added to the culture medium at a concentration of 10 μM for 3 days. LY294002, VS4718, and ZINC40099027 were administered at 2.5 μM for 7 hours, 10 μM for 7 hours, and 20 μM for 3 days, respectively. We confirmed that the vehicle controls (DMSO and distilled water) showed no detectable effects. St4n1, an antagonistic peptide of Stx4 and its control, was purchased from KNC Lab (Kobe, Japan). St4n1 is a circular peptide composed of the functional core of syntaxin4 (AIEPQK, amino acids 103–108), connected with cysteine-glycine at the N-terminus and with cysteine at the C-terminus, followed by the introduction of a disulfide bridge between the N- and C-termini. The functional core AIEPQK is found only in the Stx4 sequence among related syntaxins, and this circular peptide exhibits a membrane-impermeable nature, acting as a specific antagonist of ExStx4 (Kadono *et al.*, 2015; Kadono *et al.*, 2012). As the control peptide, a similar circular peptide composed of the same amino acids as St4n1 but arranged in a random sequence was used.

### Organ culture

Mouse egg cylinders, made of epiblast, extraembryonic ectoderm, and visceral endoderm, were surgically isolated from ICR mouse embryos at E6.0 (SLC, Hamamatsu, Japan) and placed on porous membranes (Nuclepore Track-Etch membrane, Cytiva, Tokyo, Japan). These culture assemblies were floated on medium supplemented with either an ExStx4 antagonist, one of the small-molecule inhibitors, or a FAK activator. After two days of cultivation, the size, morphology, and expression profiles of *Nanog*, *T* (*Brachyury*), and *CDH3* (*P-cadherin*) were compared with those of the control. As ExStx4 antagonists, a recombinant F3 fragment in Stx4 (rF3) prepared in bacterial strain BL21 (amino acids 198–273) (Hirose *et al.*, 2018; Shirai *et al.*, 2017), or St4n1 was added to the medium at a concentration of 20 or 1 μg/ml, respectively.

### Immunodetection

Immunoblot analyses were performed following standard protocols with minor modifications. In brief, protein bands detected by antibodies on PVDF membranes were visualized using ECL reagent (Thermo Fisher Scientific) and the ImageQuant system (GE Healthcare Japan, Tokyo, Japan). The primary antibodies used included those against T7-tag (MBL, Tokyo, Japan), P-cadherin (a generous gift from Dr. Takeichi), β-actin (Sigma-Aldrich), total FAK, total AKT, phospho-FAK, and phospho-AKT (CST Japan, Tokyo, Japan). HRP-conjugated secondary antibodies were obtained from Sigma-Aldrich and Invitrogen. To analyze the expression of Stx4 and Brachyury in the early embryo, cryosections of E6.5 egg cylinders were dual-stained with anti-Stx4 (Kadono *et al.*, 2012) and anti-brachyury (Novus Biologicals, Centennial, CO, USA) antibodies, followed by Alexa488- and Cy3-labeled secondary antibodies. To assess Rho/ROCK activation, mESCs-ExStx4 and mESCs-P-cad were cultured on gelatin-coated glass-bottom dishes (Matsunami, Kishiwada, Japan) with or without dox. Cells treated with dox were also exposed to one of the small-molecule inhibitors. After three days, cells were permeabilized with 0.05% Triton X-100, preincubated in medium containing 5% FBS, and stained with anti-phospho-myosin light chain (pMLC) antibodies (Rockland Biochem., Limerick, PA, USA) and AlexaFluor546-conjugated secondary antibodies (Invitrogen). For F-actin visualization, permeabilized cells were stained with Alexa488-labeled phalloidin (Thermo Fisher Scientific). Nuclei were counterstained with Hoechst 33258 (Dojindo, Tokyo, Japan), and images were captured using a TCS SPE system (Leica, Tokyo, Japan).

### Quantitative reverse transcription PCR (qRT-PCR)

Total RNA was extracted using the RNA Extraction Miniprep System (VIOGENE, Taipei, Taiwan) and reverse-transcribed with an RNA-PCR kit (Takara Bio, Kusatsu, Japan). The qRT-PCR reaction was performed using the Fast Start Essential DNA Green Master on the Thermal Cycler Dice (Takara Bio) or 7300 Real-Time PCR system (Applied Biosystems). The primer pairs used in this study are listed in Table 1, and the expression levels of each mRNA were normalized to that of β-actin.

### Statistical analyses

For each condition, three independent biological replicates were analyzed using the ΔΔCt method. The standard deviation (SD) was calculated from the fold changes, and P-values were determined with a Student’s two-tailed t-test, with p<0.05 indicating statistical significance. One-way ANOVA followed by Tukey’s test was employed for multiple comparisons.

## Results

### Antagonistic effects of a previously isolated fragment of Stx4

**Fig. 1:**
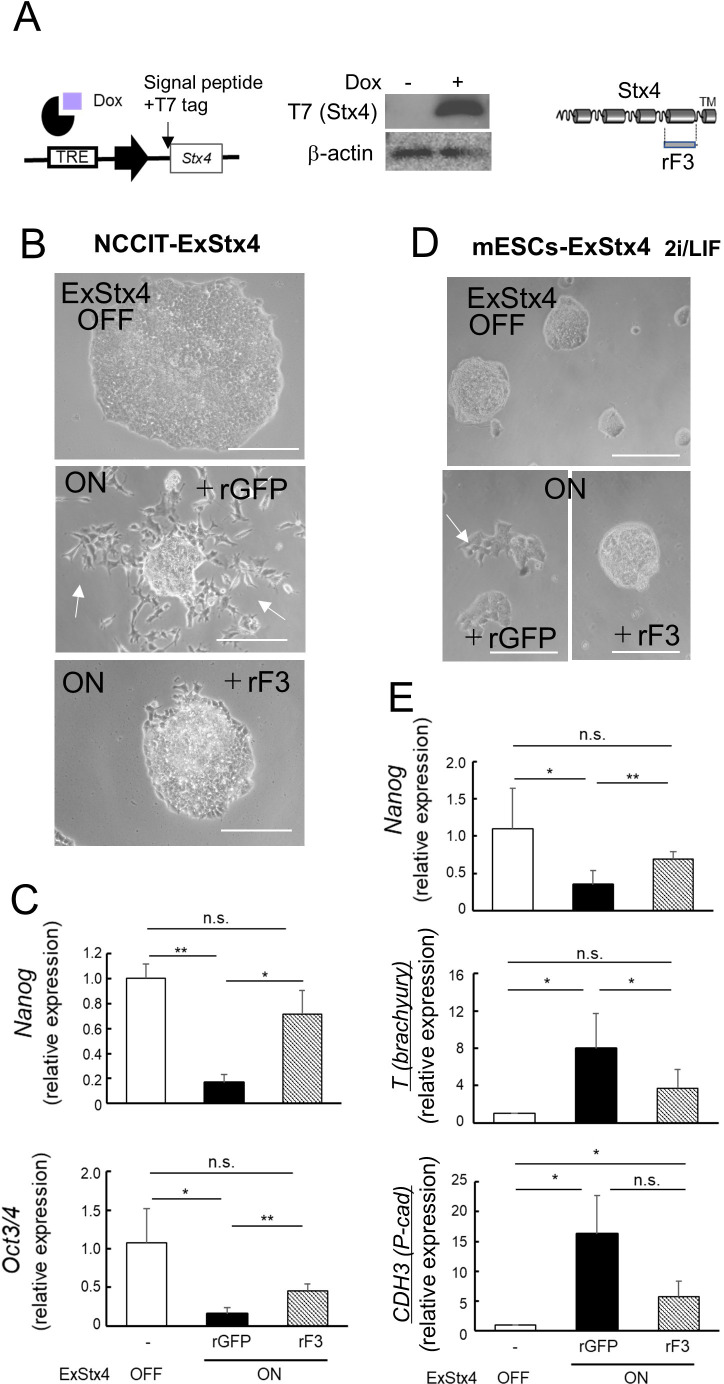
The recombinant F3 fragment (rF3) antagonizes the effect of extracellularly extruded Stx4 (A) Left, A schematic representation of the artificial expression system for ExStx4 tagged with the T7 peptide. TRE, tetracycline/doxycycline-responsive element. The Stx4 gene was fused with a signal peptide and T7-tag at the N-terminus for the efficient extracellular presentation and sensitive detection, respectively. Middle, The addition of doxycycline (dox) to the medium induces the expression of exogenous T7-tagged Stx4 at the cell surface of mESCs. Right, rF3, produced in BL21 bacterial cells, comprises the SNARE domain sequence (amino acids 198–273). (B, D) The Effects of ExStx4 with or without rF3 on the morphology of NCCIT cells (B) and mESCs (D). Bars, 100 μm. (C) The Effects of ExStx4 with or without rF3 on the expression of stemness markers *Nanog* (upper) and *Oct3/4* (lower) in NCCIT cells. (E) The Effects of ExStx4 and rF3 on the expression of *Nanog* (upper), the gastrulation marker*T* (*brachyury*) (middle), and the gastrulation onset marker *CDH3* (*P-cadherin*) (lower). Recombinant GFP (rGFP) was used as a control. n = 3, *: p<0.05, **: p<0.01.

While Stx4, a type IV membrane protein, is usually expressed on the cytoplasmic surface of the plasma membrane, it is often flipped to the cell surface in specific cell populations to perform its latent functions as a non-diffusible factor (Hagiwara-Chatani *et al.*, 2017; Kadono *et al.*, 2015; Shirai *et al.*, 2017). Since the mechanism of its membrane translocation remains unknown, we examined the antagonistic effects of rF3 using derivatives of NCCIT cells and mESCs, in which cells were introduced with a dox-inducible Stx4 expression cassette tagged with a signal peptide and T7-tag (Fig. 1A). The rF3, one of the recombinant protein fragments derived from Stx4, comprises 76 amino acids corresponding to the Stx4 SNARE domain (Fig. 1A) and exhibits potent antagonistic activity against Stx4 in mammary epithelial cells under extracellular conditions (Hirose *et al.*, 2018; Shirai *et al.*, 2017). Although we cannot completely exclude the possibility that this fragment translocates across the cell membrane to exert its function also on the cytoplasmic surface, membrane-penetrating activity of polypeptides requires characteristic arginine-rich clusters (Futaki, 2005; Nakase *et al.*, 2004), which rF3 lacks; therefore, we infer that rF3 primarily acts as an antagonist of cell surface Stx4 (ExStx4). Before the experiments, we confirmed the local signal propagation by a non-diffusible ExStx4: in mixtures of mESC-ExStx4 clones, each expressing different levels of ExStx4, P-cadherin, a downstream effector of ExStx4, was not uniformly induced but was preferentially expressed in cells with high ExStx4 expression (Supplementary Fig. S2). We found that while NCCIT cells formed well-packed colonies, and ExStx4 induced morphological changes and enhanced cell spreading, which were blocked by rF3 but not by a recombinant form of green fluorescent protein (rGFP) (Fig. 1B). In addition, ExStx4 induced the downregulation of stemness markers *Nanog* and *Oct3/4*, and this effect was reverted by rF3 (Fig. 1C, Supplementary Fig. S3). In mESCs, morphological changes caused by ExStx4 seemed to be less severe when stemness factors 2i and LIF were present (Fig. 1D) compared to conditions without 2i (Hagiwara-Chatani *et al.*, 2017). However, ExStx4 not only decreased the stemness marker *Nanog* but also increased the gastrulation marker *T* (*brachyury*) and the onset marker *CDH3* (*P-cadherin*), with the former being blocked, and both of the latter being susceptible to blockage by rF3 (Fig. 1E, Supplementary Fig. S3). These observations suggest that the extracellular presentation of Stx4 plays a key role in determining the fate of pluripotent mESCs.

### Early embryonic development and functional attenuation of extracellularly extruded Stx4

**Fig. 2:**
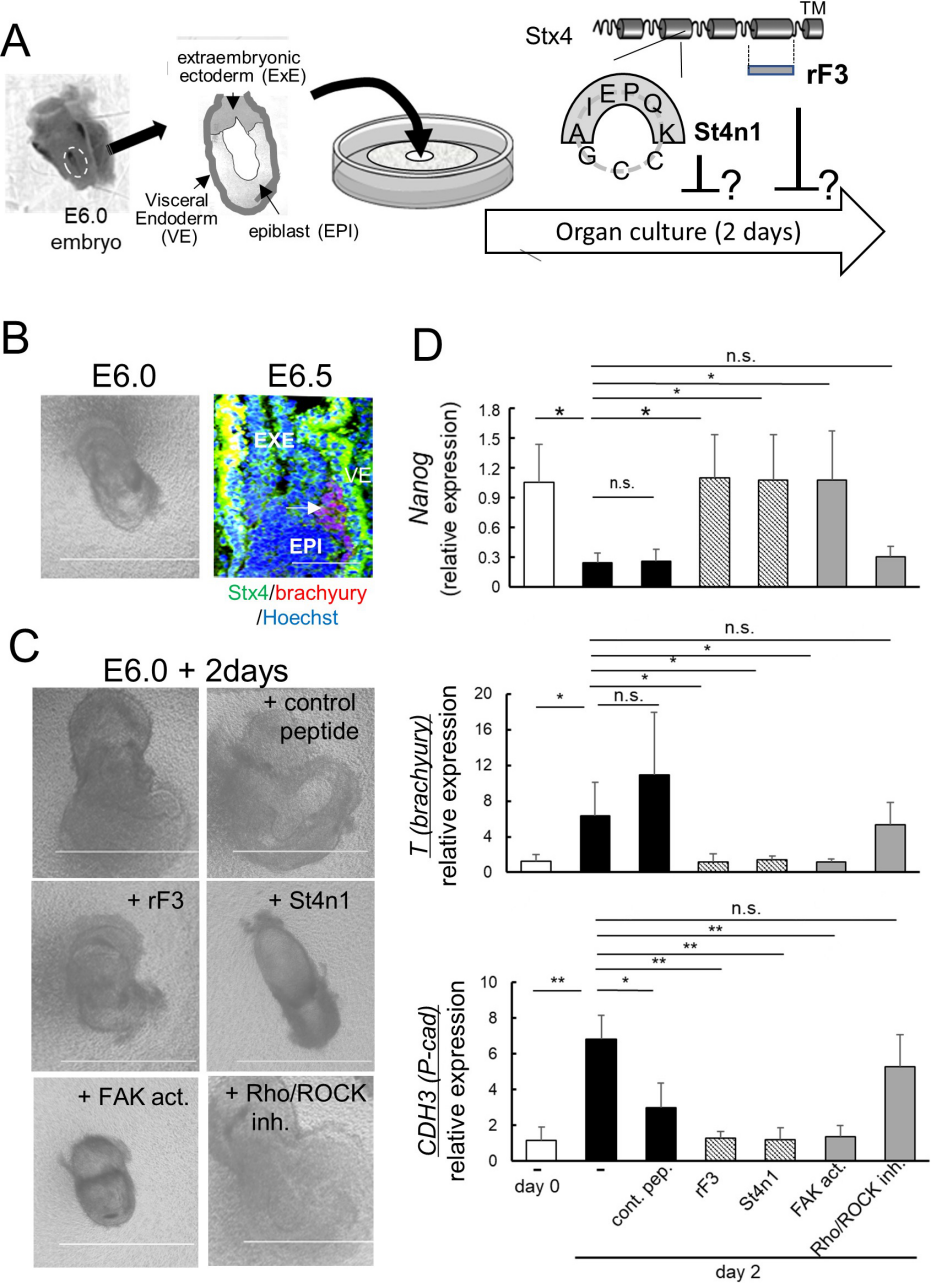
Effects of antagonists against ExStx4 and potential inhibitors of ExStx4-triggered signaling on early embryogenesis in culture (A) Left, A schematic representation of the organ culture system. Embryonic egg cylinders, comprising the epiblast (EPI), extraembryonic ectoderm (ExE), and visceral endoderm (VE), were isolated from E6.0 mouse embryos and cultured on a porous membrane floating on the medium. Right, A schematic illustration of specific ExStx4 antagonists, St4n1 and rF3. (B) Left, an egg cylinder isolated from the E6.0 embryo. Right, a cryosection of E6.5 egg cylinder that has started gastrulation (arrow). Green, Stx4. Red, brachyury, Blue, Hoechst 33258. Bars, 100 μm (left) and 500 μm (right). (C) Structure/shape of the E6.0 egg cylinders cultured for two days, in the presence or absence of rF3, St4n1, the FAK activator ZINC40099027 (FAK act.), or the Rho/ROCK inhibitor Y27632 (Rho/ROCK inh.). Bars, 500 μm. (D) Effects on the expression of the stemness marker *Nanog* (upper), the gastrulation marker *T* (*brachyury*) (middle), and the gastrulation onset marker *CDH3* (*P-cadherin*) (lower) in the absence or presence of rF3, St4n1, FAK act., or Rho/ROCK inh. Three egg cylinders were analyzed for each category. *: p<0.05, **: p<0.01.

To explore whether these findings are relevant during early mammalian development, we tested the effect of rF3 on cultured mouse early embryos (Fig. 2A). Pre-streak-stage egg cylinders from E6.0 embryos are known to initiate gastrulation within 24 hours in vivo (Beddington and Robertson, 1998; Rivera-Pérez and Hadjantonakis, 2014). Consistently, we observed brachyury-positive regions in E6.5 embryos (Fig. 2B). In culture, however, E6.0 egg cylinders rapidly migrate on the membrane in our system, where the structures and shapes of all tissue components undergo dramatic changes, thereby the epiblast, extraembryonic ectoderm, and visceral endoderm become indistinguishable within two days (Fig. 2C, Upper panels). In contrast, E6.0 egg cylinders incubated with rF3 apparently showed less change in overall size and shape (Fig. 2C, middle panels), implying the possible contribution of extruded Stx4 to cellular behaviors during early embryogenesis. Although rF3 is practically membrane impermeable, one might argue that its possible membrane translocation in this three-dimensional environment is plausible, given that epimorphin, another class of cognate syntaxin, requires the F3 (SNARE) domain for translocation across the membrane (Hirai *et al.*, 2007). We thus further examined the effect of another antagonist. In previous studies on epimorphin and Stx4, we determined that an amino acid sequence in each protein lies at the C-terminus of helix a, SIEQSC in epimorphin and the AIEKQK for Stx4, as the core for their extracellular functions (Hirai *et al.*, 2005; Kadono *et al.*, 2015; Takebe *et al.*, 2003). Among several circular peptides that expose the Stx4’s core six amino acids with different arcuation, one, termed St4n1, appeared to be readily water-soluble and explicitly antagonized ExStx4 (Kadono *et al.*, 2015). We found that St4n1, but not the control peptide, inhibited cellular migration and altered the structure of the egg cylinders, as observed in rF3-treated samples (Fig. 2C, upper and middle panels). However, both rF3 and St4n1 seemed to reduce morphological changes not only in the epiblast but also in the extra-embryonic ectoderm and visceral endoderm (Fig. 2C), implying that the effects of ExStx4 are not restricted to gastrulating cells (Fig. 2C, upper and lower panels). In line with this, Stx4 was abundantly present in all these tissues, albeit the ratio and timing of its extracellular presentation remain unclear (Fig. 2B). Importantly, however, we observed dramatic changes in the expression of stemness and gastrulation-related genes concomitant with these morphological behaviors. In control cultures, egg cylinders downregulated the stemness marker *Nanog*, and upregulated gastrulation marker *T* (*brachyury*) and the onset marker *CDH3* (*P-cadherin*), indicating that the progression of the developmental process toward gastrulation was not problematically disrupted in this culture (Fig. 2D). When supplemented with rF3 or St4n1, these cultures showed a reduction in the changes (Fig. 2D, Supplementary Fig. S3). This suggests that the extracellular presentation of endogenous Stx4 could be an essential signal for initiating gastrulation during early embryogenesis, as well. Curiously, the control peptide downregulated *CDH3*, suggesting some effects of the randomized functional core sequence of Stx4, although this had no impact on the expression of *Nanog* and *T* (Fig. 2D, lower).

### Inactivation of FAK is a key downstream mediator of ExStx4-induced effects

In NCCIT cells, ExStx4 reduces the phosphorylation of focal adhesion kinase (FAK), which subsequently activates Rho/ROCK, leading to morphological changes, and inhibits the PI3K/AKT pathway, resulting in a loss of stemness (Supplementary Fig. S1). To assess the role of FAK inactivation during early mammalian development, we also examined whether altering FAK activation states affects early mouse embryogenesis in culture. When treated with a FAK activator, ZINC 40099027, these cultures maintained their overall size, structure, and levels of the stemness marker *Nanog*, as well as the gastrulation markers *T* (*Brachyury*) and *CDH3* (*P-cadherin*), similar to what was observed in cultures treated with rF3 or St4n1 (Fig. 2C and D). This suggests that FAK inactivation may play a crucial role in the ExStx4-induced signal, even during early development. Interestingly, while FAK inactivation follows *CDH3* upregulation in NCCIT cells, the FAK activator prevented *CDH3* upregulation in the egg cylinder. Additionally, no significant effects were observed with the Rho/ROCK inhibitor Y27632, suggesting that our organ culture system may not replicate Rho/ROCK-dependent cell behaviors in gastrulation (Fig. 2C and D).

### FAK regulates the cell behaviors of mESCs

**Fig. 3:**
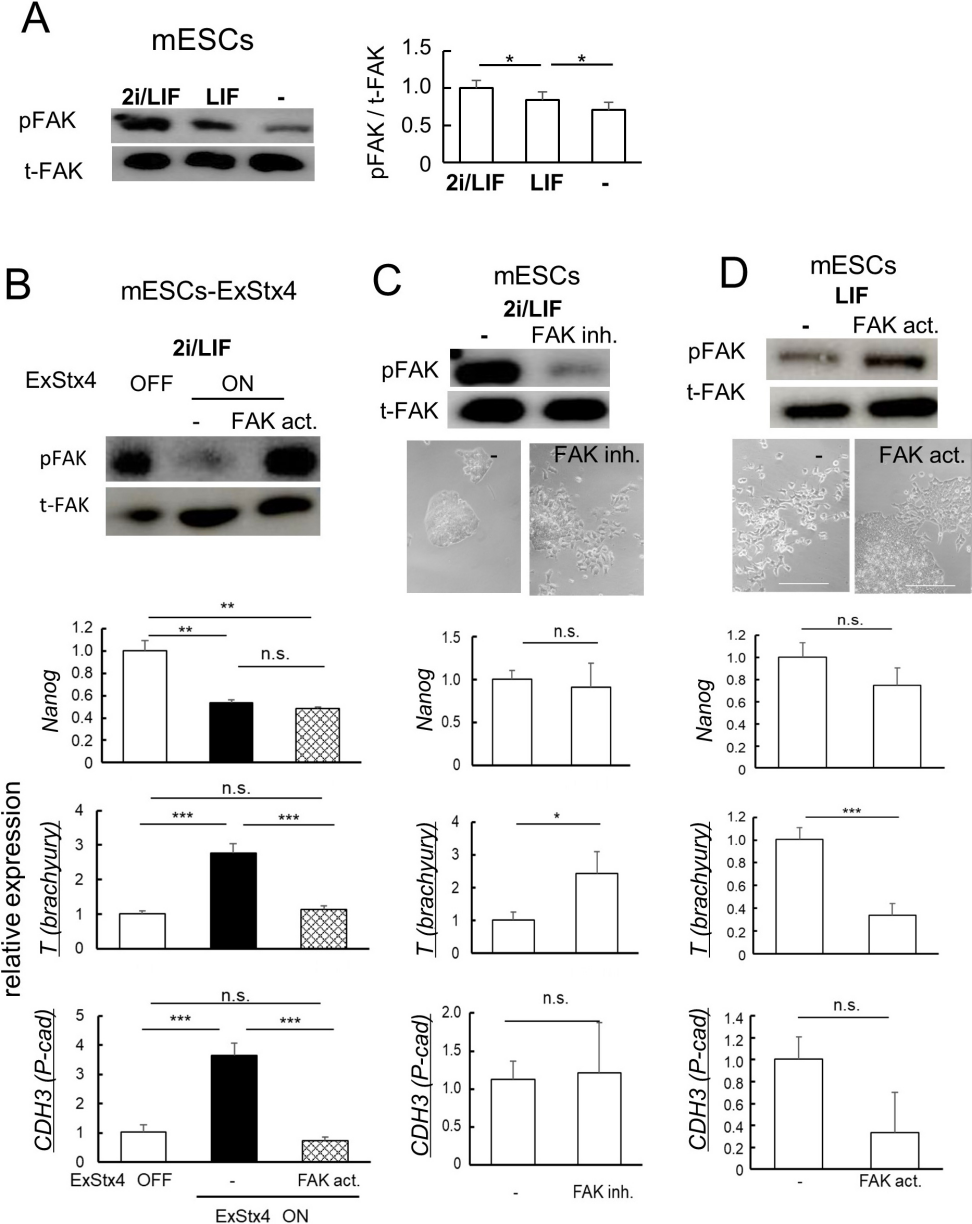
FAK inactivation as a downstream event of the ExStx4-triggered signaling pathway (A) The relative ratio of phosphorylated FAK (pFAK) to total FAK (t-FAK) in mESCs cultured with or without stemness factors. Left, Representative Western blot images. Right, Quantification. n = 3, *: p<0.05. FAK phosphorylation decreased upon removal of stemness factors. (B) Upper, Representative western blot image of pFAK and t-FAK in mESCs-ExStx4 cultured in 2i/LIF medium, with or without FAK activator ZINC40099027 (FAK act.). Lower, Quantification of relative mRNA expression of *Nanog*, *T* (*brachyury*), and *CDH3* (*P-cadherin*). n = 3, **: p<0.01, ***: p<0.001. (C, D) Upper, Amount of pFAK and t-FAK in mESCs cultured in 2i/LIF medium, with or without FAK inhibitor VS4718 (FAK inh.) (C), and in LIF-only medium with or without FAK activator ZINC40099027 (FAK act.) (D). Middle, Morphology of mESCs. Bars, 100 μm. Lower, the mRNA expression of *Nanog*, *T* (*brachyury*), and *CDH3* (*P-cadherin*). n = 3, *: p<0.05, ***: p<0.001.

Considering the potential role of FAK inactivation in ExStx4-induced signals, we then examined how FAK phosphorylation affects the behaviors of pluripotent stem cells, using mESCs, which are derived from the inner cell mass of blastocysts. First, we measured phosphorylated FAK levels in three different media: with or without the stemness factors 2i and/or LIF, to maintain or reduce an undifferentiated state. In mESCs, FAK inactivation was evident during spontaneous differentiation (Fig. 3A), similar to what was observed in cells expressing ExStx4 (Fig. 3B, upper). We found that ExStx4-induced FAK inactivation was accompanied by significant upregulation of the gastrulation markers *T* (*Brachyury*) and *CDH3* (*P-cadherin*), both of which were blocked entirely by FAK activator ZINC 40099027 (Fig. 3B, lower panels, Supplementary Fig. S3). Notably, however, ExStx4-induced downregulation of the stemness marker *Nanog* was not protected by this FAK activator (Fig. 3B, middle). In mESCs cultured with stemness factors 2i and LIF, pharmacological FAK inhibition with VS4718 caused morphological changes and increased the expression of *T*, a gastrulation marker, as observed in Stx4-expressing mESCs, mESCs-Stx4 (Fig. 3C, Supplementary Fig. S3). Conversely, in medium containing LIF but lacking 2i, which promotes a subset of cells to extrude endogenous Stx4 at the cell surface (Hagiwara-Chatani *et al.*, 2017) and decreases FAK phosphorylation (Fig. 3A), both the morphological changes and the upregulation of *T* were prevented by the FAK activator (Fig. 3D, Supplementary Fig. S3). While the expression of *Nanog* and *CDH3* in mESCs was not influenced by artificial modulation of FAK activity (Fig. 3C and D), the suppression of *CDH3* expression by the FAK activator became apparent when both 2i and LIF were withdrawn, conditions that facilitate the extrusion of endogenous Stx4 and promote spontaneous differentiation (Supplementary Fig. S4). These results suggest that FAK inactivation is an upstream event that leads to the upregulation of P-cadherin and partly mediates the effects of ExStx4 in mESCs, similar to what occurs during early embryogenesis.

### Analysis of downstream mediators of extracellular Stx4 in mESCs

**Fig. 4:**
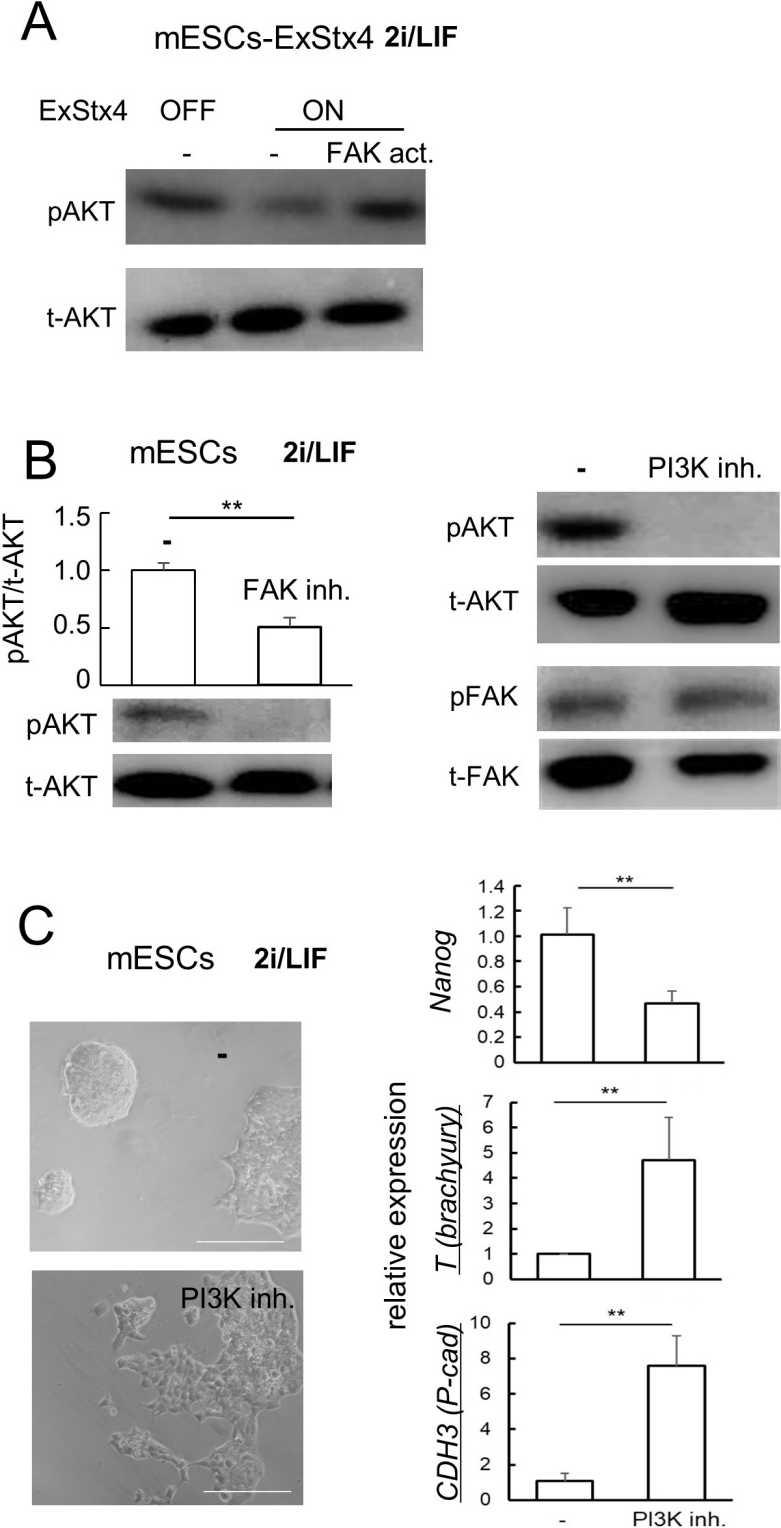
PI3K/AKT inactivation as a critical downstream component of ExStx4-induced FAK inactivation (A) Effects of ExStx4 with or without the FAK activator (FAK act.) on the phosphorylation level of PI3K/AKT. pAKT, phosphorylated AKT. tAKT, total AKT. (B) Left, The effect of the FAK inhibitor VS4718 (FAK inh.) on AKT phosphorylation in mESCs. n = 3, **: p<0.01. Right: The effect of the PI3K/AKT inhibitor LY294002 on FAK phosphorylation. (C) The effect of the PI3K/AKT inhibitor on the phenotypic appearance (left) and the expression of *Nanog*, *T*, and *CDH3* in mESCs. Bars on the left, 100 μm. Right, n = 3, **: p<0.01.

In NCCIT cells, ExStx4-induced FAK inactivation led to the suppression of PI3K/Akt signaling. Similarly, in mESCs-Stx4, the induced expression of ExStx4, which caused FAK inactivation (Fig. 3 B), decreased the phosphorylation of AKT, and this was prevented by the FAK activator (Fig. 4A). Based on this, we examined the functional relationship between FAK and PI3K in parental mESCs. While inhibiting FAK decreased the phosphorylated level of AKT, inhibiting PI3K/AKT did not affect the phosphorylated level of FAK (Fig. 4B), indicating that FAK is upstream of PI3K/AKT in the ExStx4-triggered signaling pathway. Additionally, inhibiting AKT significantly increased *T* and *CDH3* levels, while decreasing *Nanog* expression (Fig. 4C, Supplementary Fig. S3). These findings suggest a Stx4-FAK-PI3K-P-cadherin axis: ExStx4-induced FAK inactivation influences morphological changes and differentiation through PI3K/Akt inactivation, leading to increased P-cadherin expression in mESCs. This response differs from what was observed in NCCIT cells. While PI3K/AKT signaling primarily regulates differentiation in NCCIT cells (Nozaki and Hirai, 2023), the AKT inhibitor also affected morphological changes in ESCs (Fig. 4C), suggesting that PI3K/AKT may have a distinct role in the signaling pathway triggered by ExStx4.

### Importance of P-cadherin expression in ExStx4-induced ESCs behavior

**Fig. 5:**
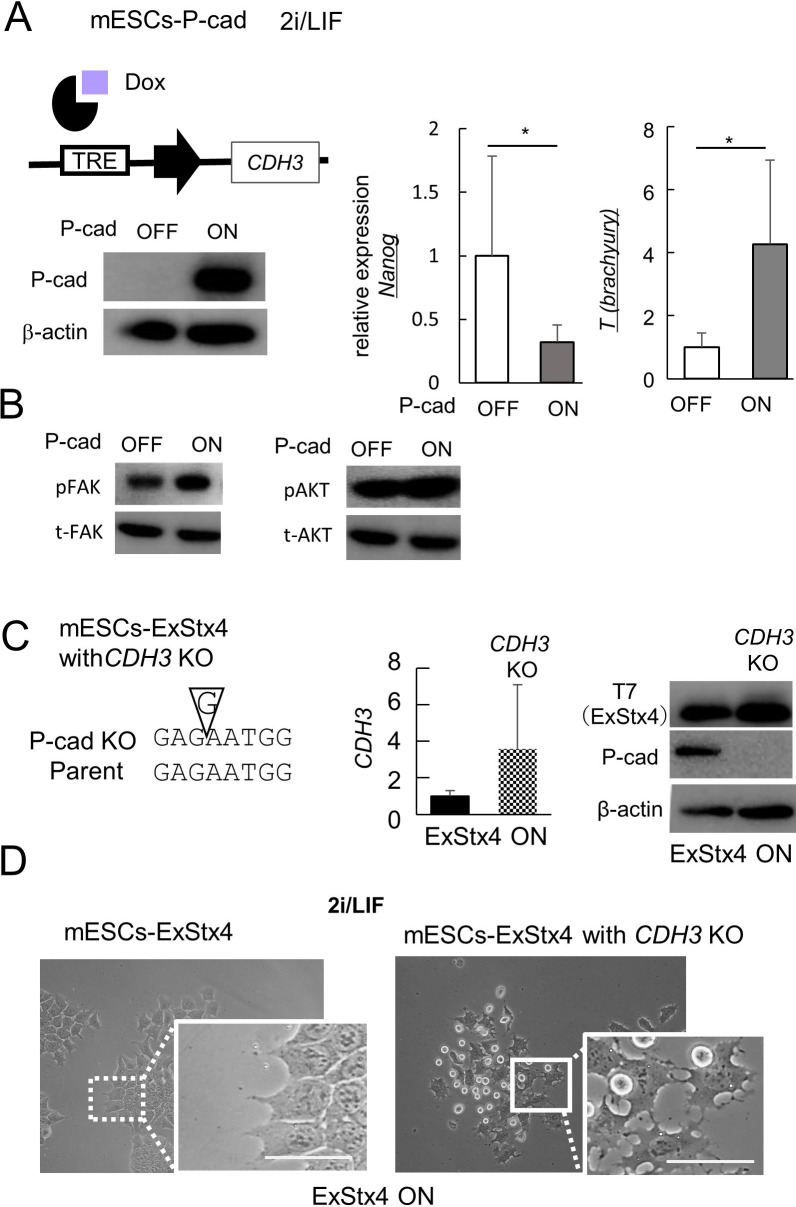
Upregulation of P-cadherin as a downstream component of the ExStx4–FAK–PI3K signaling axis (A) Left, A schematic representation of the dox-inducible expression system for P-cadherin in mESCs (mESCs-P-cad). Right, Effect of P-cadherin expression on the expression of *Nanog* and *T* *(brachyury)*. n = 3, *: p<0.05. (B) Effects of P-cadherin expression on the phosphorylation levels of FAK or AKT. (C) P-cadherin knockout (KO) in mESCs-ExStx4 cells. Left, a guanine insertion caused a frameshift mutation in the CDH3 gene. Middle and right, the effect of P-cadherin KO on the expression of *CDH3* mRNA (middle) and P-cadherin protein (right). β-actin was used as a loading control. (D) Phenotypic appearances of ESCs-ExStx4 expressing ExStx4 with (right) or without (left) P-cadherin KO. Inlets, enlarged images. Bars, 20 μm.

We then clarify the potential role of P-cadherin, whose expression increases with ExStx4 expression. A previous study demonstrated that the exogenous introduction of P-cadherin produced similar ESC behaviors during 2i removal and in LIF-containing medium, as observed with ExStx4. ESCs with either exogenous ExStx4 or P-cadherin exhibited a flattened shape, along with signs of differentiation toward gastrulation (Matsuguchi and Hirai, 2023; Takeda *et al.*, 2023). In 2i/LIF medium, inducing exogenous P-cadherin resulted in the downregulation of the stemness marker *Nanog* and the upregulation of the gastrulation marker *T*, similar to the effects of ExStx4 (Fig. 5A). As expected, the phosphorylation levels of FAK and AKT, which both decreased upon ExStx4 expression, remained unchanged with P-cadherin expression, confirming that the inactivation of FAK and PI3K/AKT are upstream events in ExStx4-triggered P-cadherin upregulation (Fig. 5B). To understand P-cadherin’s role in ExStx4-triggered signaling better, we introduced a frameshift mutation into the P-cadherin gene (*CDH3*) in mESCs-ExStx4, which harbor a dox-inducible ExStx4 transgene. We isolated several clones that express *CDH3* mRNA but not P-cadherin protein (Fig. 5C). The resulting P-cadherin-deficient mESCs, despite expressing ExStx4 by dox-treatment, showed different morphology compared to the original ExStx4-expressing cells, with some failing to adhere to the substrate and others losing cell–cell adhesion (Fig. 5D). These findings confirm that P-cadherin upregulation also contributes to the responses caused by ExStx4 in ESCs.

### Rho/ROCK activation in ExStx4-induced morphological changes in ESCs

**Fig. 6:**
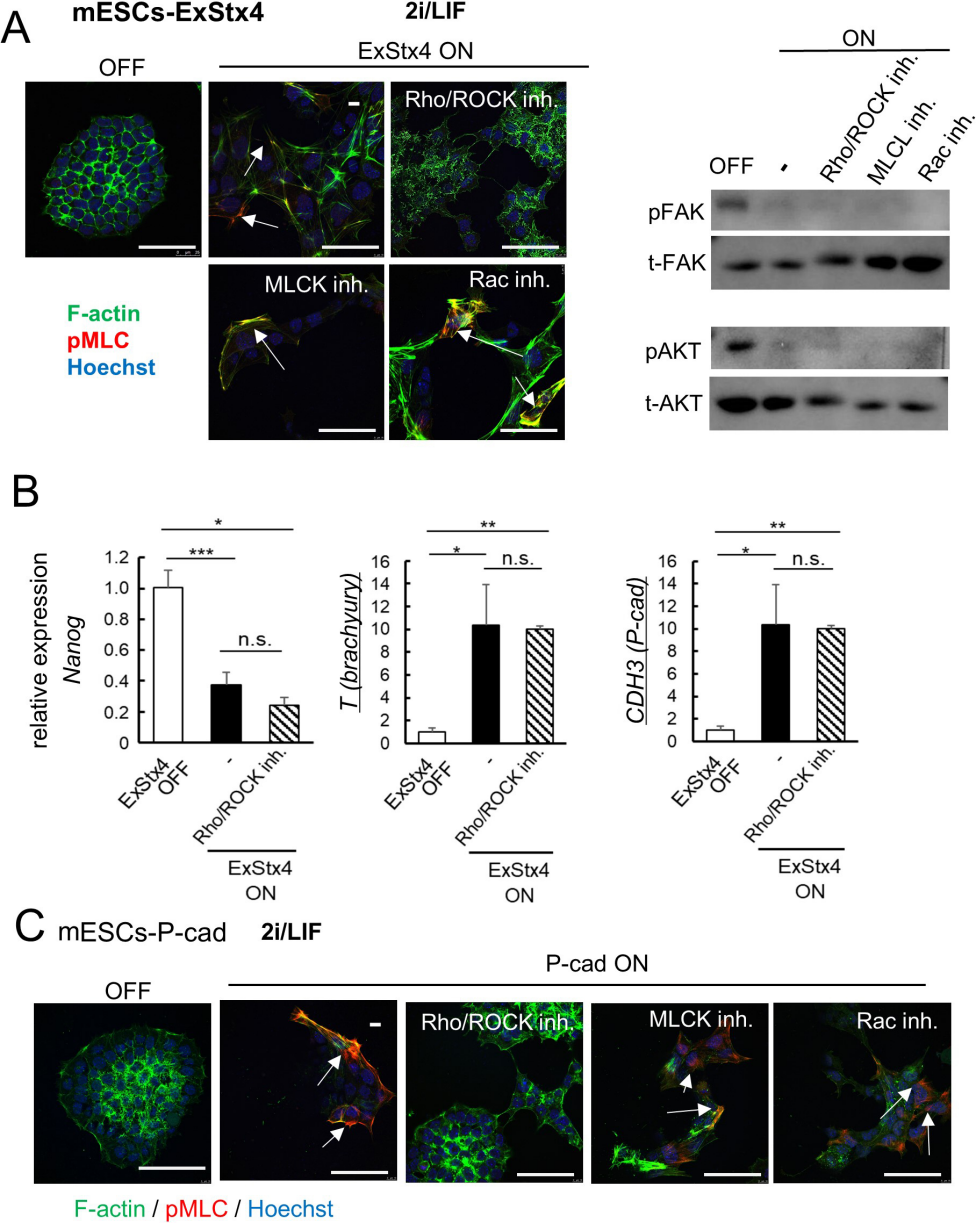
ExStx4/P-cadherin signaling leads to Rho/ROCK activation and alters F-actin arrangement (A) Left, Effects of the Rho/ROCK inhibitor Y27632, but not by the MLCK inhibitor ML-7 or the Rac inhibitor NSC23766, on ExStx4-induced morphological changes visualized by F-actin staining. Green, F-actin. Red, phosphorylated MLC (pMLC). Nuclei were counterstained with Hoechst 33258 (blue). Arrows indicate areas of Rho/ROCK activation, visualized by pMLC staining. Note that cells treated with ML-7 exhibited Rho/ROCK-induced MLC phosphorylation, as ML-7 inhibits pMLC activity but not its phosphorylation. Bars, 60 μm. Right, Effects of the inhibitors used in the left panels on ExStx4-induced inactivation of FAK and AKT. (B) Effects of the Rho/ROCK inhibitor on ExStx4-induced changes in the expression of *Nanog*, *T*, or *CDH3*. n = 3, *: p<0.05, **: p<0.01, ***: p<0.001. (C) Effects of inhibitors shown in (A) on P-cadherin-induced morphological changes, as visualized by F-actin staining. Arrows, Rho/ROCK-activated regions. Bars, 60 μm.

In NCCIT cells, extracellular Stx4 induces morphological changes associated with actin reorganization, transitioning from stress fibers to actin belts, via the activation of Rho/ROCK (Supplementary Fig. S1). In mESCs-ExStx4, the Rho/ROCK inhibitor Y27632, but not the Rac inhibitor NSC or the MLCK inhibitor ML-7, was able to rescue these morphological changes with F-actin rearrangements (Fig. 6A, Left). However, none of these inhibitors prevented the ExStx4-induced inactivation of FAK and PI3K/AKT (Fig. 6A, right), indicating that Rho/ROCK activation is the downstream effector in the ExStx4-FAK-PI3K/AKT pathway. Conversely, this Rho/ROCK inhibitor did not block ExStx4-induced changes in the expression of *Nanog*, *T*, and *CDH3*, suggesting that the morphological changes driven by Rho/ROCK activation are the distinct events for differentiation and brachyury expression (Fig. 6B, Supplementary Fig. S3). Similar to its effects on ExStx4-induced morphological effects, this inhibitor affected the morphology of P-cadherin-expressing mESCs-Pcad, indicating that Rho/ROCK activation is a downstream step of ExStx4-induced P-cadherin upregulation (Fig. 6C).

**Fig. 7:**
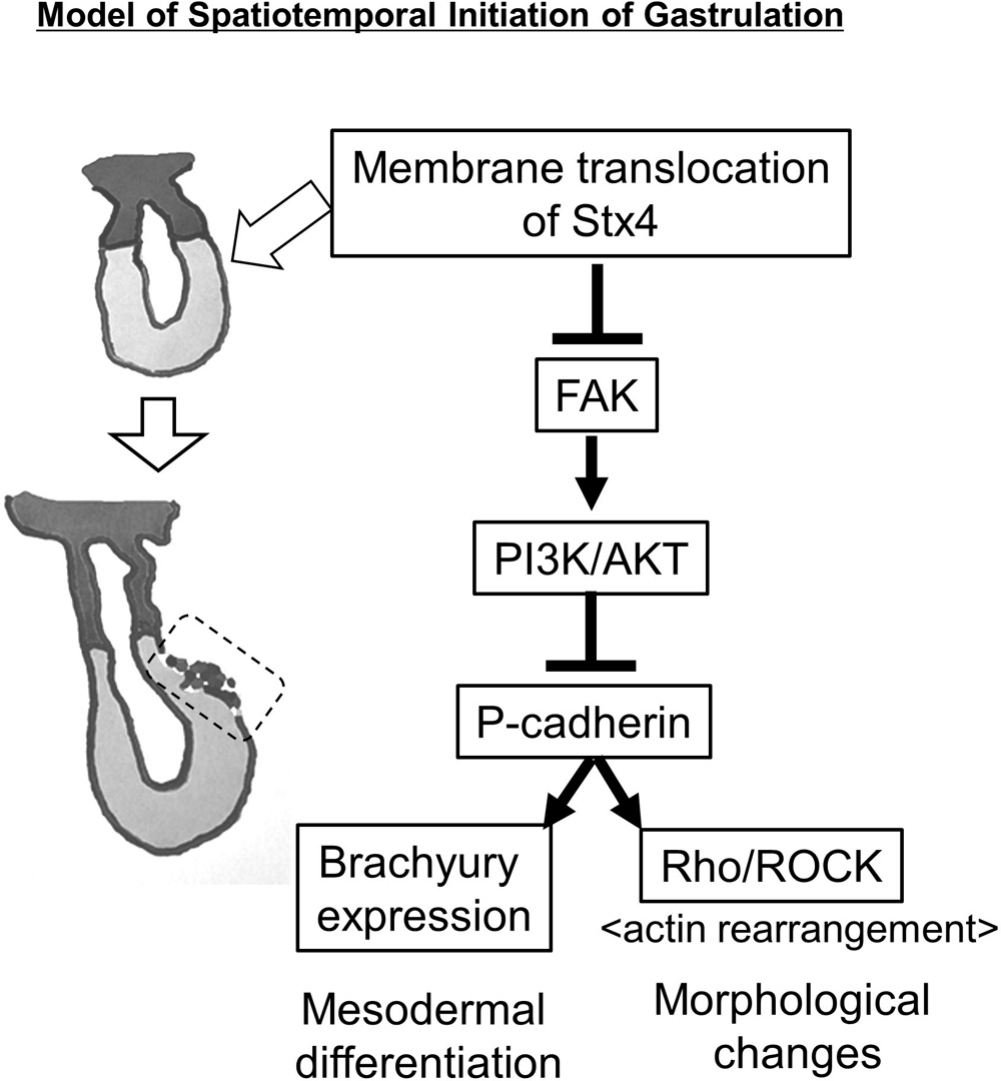
A model for the spatiotemporal initiation of gastrulation in a uniform pluripotent cell sheet Left, Schematic representation of gastrulation in the early mouse embryo. Right, A proposed molecular mechanism underlying the spatiotemporal initiation of gastrulation. Stx4, a type IV membrane protein, translocates to the cell surface in a spatially restricted region and activates a latent signaling pathway.

Taking these observations together, extracellular Stx4 may play a role in early developmental processes, such as region-specific gastrulation: ExStx4 inactivates FAK and subsequently PI3K/AKT, leading to the upregulation of P-cadherin, which then triggers the loss of stemness and upregulates gastrulation-specific Brachyury, along with Rho/ROCK-dependent morphological changes (Fig. 7).

## Discussion

As a pivotal event in early embryogenesis, gastrulation has garnered significant attention, prompting researchers to thoroughly investigate its underlying molecular mechanisms. Although it is now understood that cells undergoing gastrulation lose epithelial characteristics, reprogram transcription factor expression, and change their motility, the molecular basis for the localized initiation of this process remains elusive. We identified the membrane translocation of a type IV membrane protein, Stx4, as a potential spatiotemporal signal for triggering gastrulation, where extracellular Stx4 locally activates a unique signaling pathway that drives the progression of this event.

Using organ culture of embryonic egg cylinders isolated from E6.0 embryos, where changes in the cell environment for during gastrulation are detectable in vitro, we demonstrate that distinct forms of specific antagonists of ExStx4 block changes in the expression profiles of genes related to stemness- and gastrulation, suggesting that the extracellular extrusion of Stx4 in specific cells may trigger cell fate transitions necessary for gastrulation. One antagonist used in this study, a recombinant peptide fragment of Stx4, rF3, competitively binds to E-cadherin and laminin, known binding molecules of Stx4 (Hirose and Hirai, 2021; Shirai *et al.*, 2017). Another antagonist was the circular peptide generated from the functional core of extracellularly extruded Stx4 (Kadono *et al.*, 2015). The system’s validity was further confirmed by observing that artificial activation of focal adhesion kinase (FAK), which inhibits ExStx4-induced FAK inactivation, also suppressed ExStx4-induced cellular behaviors. FAK, a non-receptor protein tyrosine kinase, mediates signaling triggered by interactions between cell surface integrins and extracellular matrix (ECM) components (Peng and Guan, 2011). In the epiblast, gastrulating cells induce substantial remodeling of the underlying ECM (Nakaya and Sheng, 2009), and FAK is locally regulated during this process (Zhang *et al.*, 1995). Although information on the local activity of FAK in the epiblast at the onset of gastrulation remains limited, previous studies reported a region-specific reduction in both the expression and phosphorylation of FAK within the gastrulation area (Ridyard and Sanders, 1998), and genetic ablation of this kinase did not affect the initiation of gastrulation (Furuta *et al.*, 1995). While FAK is required after the later stages of gastrulation for embryogenesis (Furuta *et al.*, 1995; Peng and Guan, 2011), our findings on the necessity of transient FAK inactivation for gastrulation initiation are consistent with these observations. Notably, however, both the antagonists against ExStx4 and the FAK activator affected not only epiblast cell fate but also the overall size/shape of the egg cylinders. While gene expression changes related to stemness and gastrulation were limited to the epiblast, this suggests the presence and potential role of ExStx4 in neighboring tissues. Indeed, we detected abundant expression of Stx4 not only in epiblast but also in extraembryonic ectoderm and visceral endoderm. The extraembryonic ectoderm derives from the trophoectoderm of the blastocyst, which abundantly expresses Stx4 (Wyman *et al.*, 2003), Presumably, its subpopulation may also be exposed extracellularly, contributing to early embryonic development. However, identifying the specific cell population responsible for Stx4 extrusion was unsuccessful: while we attempted to label the particular cells expressing ExStx4 in living egg cylinders with anti-Stx4 antibodies, complete removal of unbound antibodies from the three-dimensional samples was not feasible.

Although ExStx4-induced Rho/ROCK activation causes morphological changes in both NCCIT cells and mESCs, a ROCK inhibitor did not affect the changes in the overall size, shape, and expressions of gastrulation-related genes in cultured egg cylinders, implying that Rho/ROCK activation might not play a role during gastrulation. However, it is possible that our organ culture system could not replicate Rho/ROCK-dependent cell behaviors in gastrulation, as previous studies have shown that the same ROCK inhibitor significantly impairs gastrulation in both Xenopus and rabbit embryos (Kim *et al.*, 2015; Stankova *et al.*, 2015). Additionally, the ROCK inhibitor has also been reported to induce epithelial-mesenchymal transition (EMT) in human ESC cultures, which is a crucial cell behavior for gastrulation (Maldonado *et al.*, 2016). An alternative possibility is that the amount of Y27632 used in this study (10 μM) might not be enough to prevent Rho/ROCK signals in three-dimensional (3D) egg cylinders, as a higher amount of this inhibitor (40 or 50 μM) was reportedly required to exert inhibitory effects in 3D blastocyst culture (Stankova *et al.*, 2015). Thus, for the accurate determination of the specific role of Rho/ROCK activation in gastrulation, it would be necessary to reinvestigate using a more refined culture system, such as those previously established (Ma *et al.*, 2019; Rossant and Tam, 2022), with a higher concentration of the inhibitor.

Although the components of ExStx4 signaling appear to be practically conserved between the epiblast/ESCs and the highly stable NCCIT cells, experiments using specific inhibitors and an activator suggest that the hierarchical relationships among downstream effectors might differ. For example, FAK inactivation occurs downstream of P-cadherin upregulation in NCCIT cells. In contrast, this relationship appears reversed in early embryogenesis, albeit both FAK inactivation and P-cadherin upregulation result in similar cellular behaviors. It is well known that P-cadherin primarily supports cell-cell adhesion but can also promote cell scattering, an effect opposite to intercellular adhesion, under certain conditions (Ma *et al.*, 2025; Sun *et al.*, 2011; Vieira and Paredes, 2015). Additionally, FAK inactivation is known to be strongly associated with changes in integrin-substrate interactions that regulate cell scattering (Jiang *et al.*, 2020; Katoh, 2020) . Given the complex cross-talk between cadherin-catenin and integrin-FAK pathways (Canel *et al.*, 2013; DeMali *et al.*, 2014; Mui *et al.*, 2016), this cell-type-specific response may explain the reversed causal relationship observed. Another possible cause for the difference is the stability of these cell types in culture. While NCCIT cells can differentiate into all three germ layers, they are very stable under standard conditions and may have lost some regulatory machinery in this signaling pathway during immortalization. In contrast, embryonic epiblast cells and ESCs depend on stemness-supporting factors such as LIF and 2i, whose downstream signals could unexpectedly influence the relationship between FAK inactivation and P-cadherin upregulation.

A key unresolved issue is how the extracellular extrusion of Stx4 is spatially restricted within the epiblast to trigger gastrulation in specific regions of stem cells. Although this mechanism is not understood, studies on skin keratinocytes have shown that calcium influx triggers the extracellular extrusion of Stx4 (Kadono *et al.*, 2015). In ESCs, artificially increasing intracellular calcium levels reportedly promotes differentiation, which is accompanied by a decrease in stemness markers (Meyer *et al.*, 2009). Additionally, a localized increase in cytoplasmic calcium has been observed in the dorsal area of the presumptive ectoderm (Hayashi *et al.*, 2018; Shindo *et al.*, 2010), and calcium sparks are known to be generated transiently in individual cells (Shirakawa *et al.*, 2016). These findings indicate the possibility that the transient calcium spark may serve as a possible cue for the targeted membrane flip of Stx4.

In conclusion, due to the non-diffusible nature and inductive ability of gastrulation-related genes in ExStx4, we propose that the membrane translocation of this molecule may play a crucial role in initiating region-specific gastrulation, a vital process in early embryonic development. We also identified the components involved in ExStx4-triggered signaling: this molecule activates the latent signaling pathway, which includes the inactivation of FAK and PI3K/AKT, as well as the upregulation of P-cadherin. This signaling ultimately promotes mesodermal differentiation through unidentified mediators and explicitly activates the Rho/ROCK pathway, leading to morphological changes.

## Funding

Part of this work is supported by JSPS KAKENHI (25KJ2236) (to SN) and KG Special research fund (to YH).

## Conflict of Interest

The authors declare no relevant financial or non-financial interests.

## Data Availability Statement

The datasets and materials used for the current study are available from the corresponding author upon reasonable request.

## Author Contributions

SN: Investigation, Methodology, Formal analysis, Validation, Data curation, Writing-initial draft; TM: Investigation; YH: Conceptation, Supervision, Writing-final draft

## Ethics Approval

This study was conducted in accordance with the principles outlined in the Declaration of Helsinki. All experiments involving animals were approved by the Animal Experiment Committee at Kwansei Gakuin University (approval number: 2023-30).

## Figures and Tables

**Table 1:** Primer pairs used for qRT-PCR analyses

*β-actin*	CCTCACCCTCCCAAAAGC	GTGGACTCAGGGCATGGA
*Oct 3/4*	AGCACTTCTGTCATGCTGGA	AGCACCTTCTATAAGCCAGCG
*Nanog*	TTCTTGCTTACAAGGGTCTGC	CAGGGCTGCCTTGAAGAG
*CDH3 (P-cad)*	GCACTGCTGACCCTTCTACTG	GGGCTCTTTGACCTTCCTC
*T (Brachyury)*	CAAGAACGGCAGGAGGATGT	ATTTCCAGCGGTGGTTGTCA
