# Supplementary material for: Extracellular presentation of syntaxin4 as a potential trigger for region-specific gastrulation
Source: Cell Struct Funct. 2025 Oct 29;50(2):197–212. doi: 10.1247/csf.25073 (PMC12967521; doi:10.1247/csf.25073)
Supplement: Supplementary file 1 — Supplementary Materials [file csf_50_25073_1.pdf]

Human embryonic carcinoma cell line NCCIT

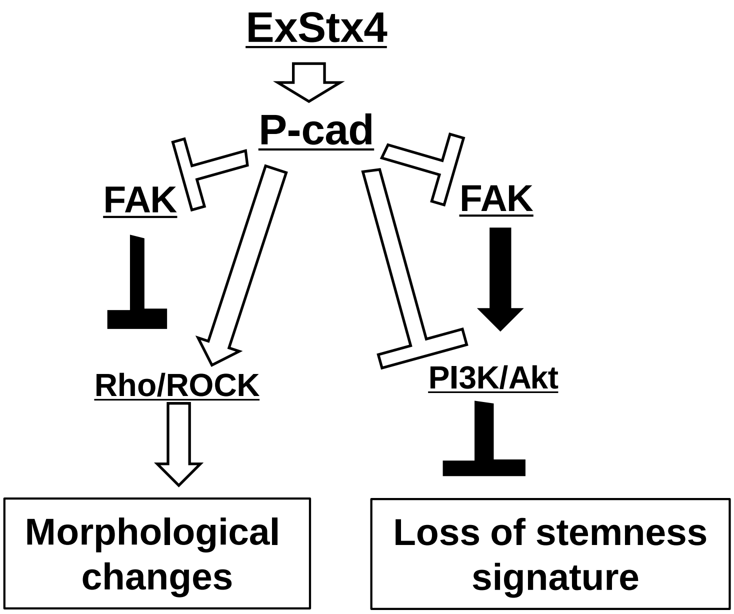

Key elements involved in morphological changes and the loss of stemness signatures induced by ExStx4 in NCCIT cells. Pathways identified through the use of ExStx4/P-cadherin-expressing NCCIT cells are shown with open arrows and open inhibitory arrows. In contrast, pathways identified through the parental NCCIT cells are shown with closed arrows and closed inhibitory arrows.

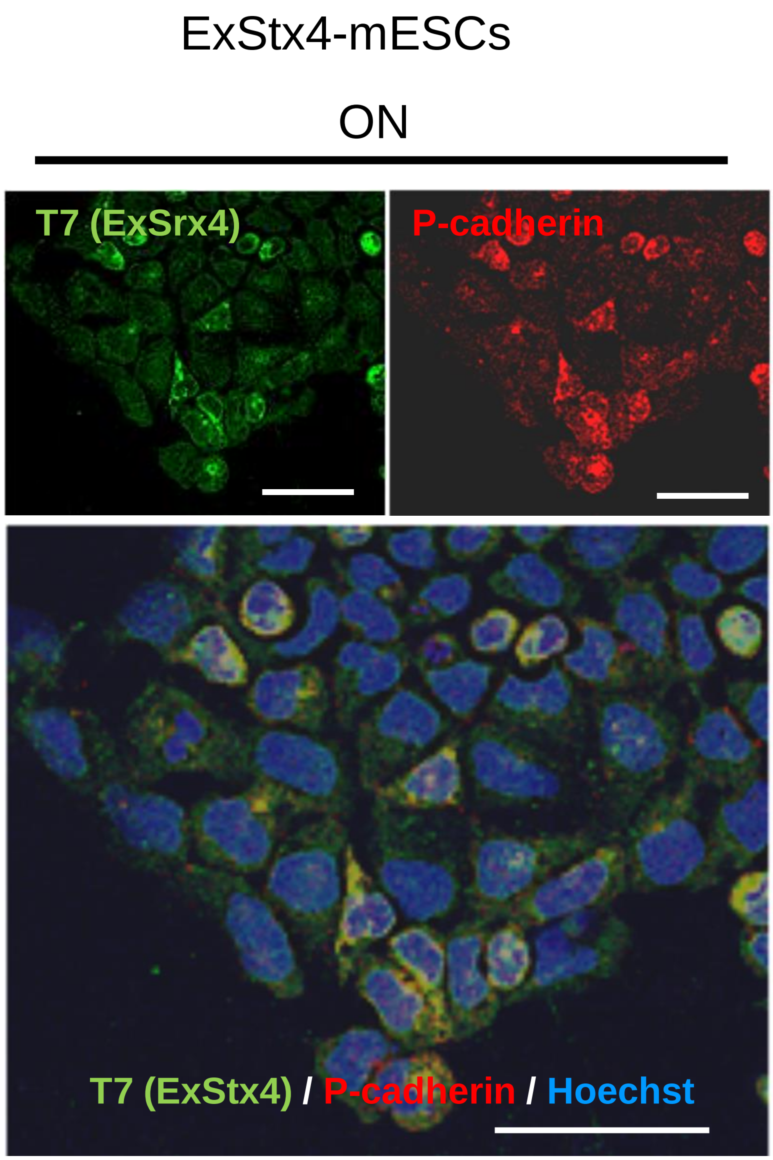

Extracellularly expressed Stx4 locally activates signals for the expression of P-cadherin. The mixture of ExStx4-mESC clones was stained for T7 tag (ExStx4, green) and P-cadherin (red). Cells were counterstained with Hoechst (blue). Bar, 50 μm. Expression levels of ExStx4 varied among the mixture of ExStx4-mESC clones, and P-cadherin was not uniformly induced across all cell populations. Instead, higher expression was observed in and around high producers of ExStx4, indicating that ExStx4 acts as a non-diffusible protein.

Raw data and statistical analysis for qRT-PCR

- Fig. 1C
- Fig. 1E
- Fig. 2D
- Fig. 3B
- Fig. 3C
- Fig. 3D
- Fig. 4C
- Fig. 6B

Raw data and statistical analysis for Fig. 1C

Target Name	Sample Name	Ct(SDM)	ΔCt
Nanog	OFF 1	22.76	6.02
	OFF 2	22.09	5.72
	OFF 3	21.98	5.75
	ON rGFP-1	24.04	8.09
	ON rGFP-2	25.02	9.22
	ON rGFP-3	24.81	8.15
	ON rF3-1	23.01	6.38
	ON rF3-2	22.9	5.95
	ON rF3-3	23.19	6.71
Oct3/4	OFF 1	20.03	3.29
	OFF 2	19.89	3.52
	OFF 3	20.83	4.6
	ON rGFP-1	22.98	7.03
	ON rGFP-2	22.67	6.87
	ON rGFP-3	22.48	5.82
	ON rF3-1	21.94	5.31
	ON rF3-2	21.78	4.83
	ON rF3-3	21.21	4.73
β-actin	OFF 1	16.74	
	OFF 2	16.37	
	OFF 3	16.23	
	ON rGFP-1	15.95	
	ON rGFP-2	15.8	
	ON rGFP-3	16.66	
	ON rF3-1	16.63	
	ON rF3-2	16.95	
	ON rF3-3	16.48	

ave Δct	ΔΔct	2^(-ΔΔCt)	ave fold change	t test	t test	sd
5.83	0.19	0.88	1.00			0.11
	-0.11	1.08				
	-0.08	1.06				
	2.26	0.21	0.17	0.006		0.06
	3.39	0.10				
	2.32	0.20				
	0.55	0.68	0.72	0.125	0.031	0.19
	0.12	0.92				
	0.88	0.54				
3.80	-0.51	1.43	1.07			0.44
	-0.28	1.22				
	0.80	0.58				
	3.23	0.11	0.16	0.046		0.08
	3.07	0.12				
	2.02	0.25				
	1.51	0.35	0.46	0.177	0.016	0.09
	1.03	0.49				
	0.93	0.53				

Analysis of each experimental group

	Target Name	Sample Name	Ct(SDM)	ΔCt	Δ Δ Ct	2^(- Δ Δ Ct)
Group 1	Nanog	OFF1	22.76	6.02	0	1
		ON rGFP-1	24.04	8.09	2.07	0.24
		ON rF3-1	23.01	6.38	0.36	0.78
Group 2	Nanog	OFF2	22.09	5.72	0	1
		ON rGFP-2	25.02	9.22	3.5	0.088
		ON rF3-2	22.9	5.95	0.23	0.85
Group 3	Nanog	OFF3	21.98	5.75	0	1
		ON rGFP-3	24.81	8.15	2.4	0.19
		ON rF3-3	23.19	6.71	0.96	0.51
Group 1	Oct3/4	OFF1	20.03	3.29	0	1
		ON rGFP-1	22.98	7.03	3.74	0.075
		ON rF3-1	21.94	5.31	2.02	0.25
Group 2	Oct3/4	OFF2	19.89	3.52	0	1
		ON rGFP-2	22.67	6.87	3.35	0.098
		ON rF3-2	21.78	4.83	1.31	0.40
Group 3	Oct3/4	OFF3	20.83	4.6	0	1
		ON rGFP-3	22.48	5.82	1.22	0.43
		ON rF3-3	21.21	4.73	0.13	0.91

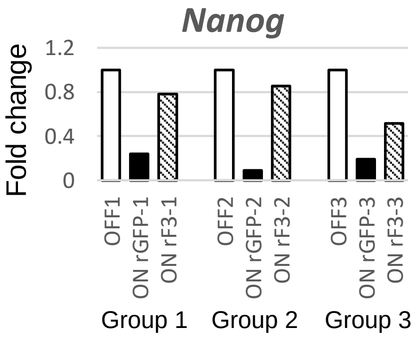

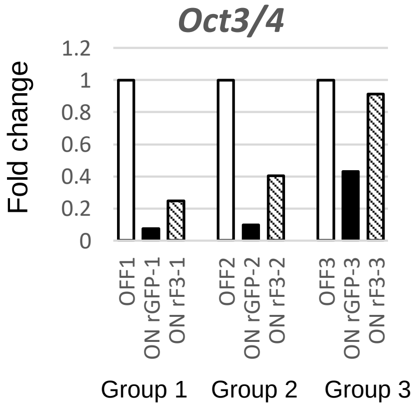

Raw data and statistical analysis for Fig. 1E

Target Name	Sample Name	Ct(SDM)	ΔCt	ave Δct	ΔΔct	2^(-ΔΔCt)	ave fold change	v.s. - t test	v.s. rGFP t test	sd
Nanog	OFF 1	22.99	3.48	2.68	0.80	0.57	1.10			0.55
	OFF 2	22.26	2.62		-0.06	1.04				
	OFF 3	22.25	1.94		-0.74	1.67				
	ON rGFP-1	24.23	5.18		2.50	0.18	0.35	0.036		0.18
	ON rGFP-2	22.70	4.27		1.59	0.33				
	ON rGFP-3	23.66	3.56		0.88	0.54				
	ON rF3-1	23.48	3.46		0.78	0.58	0.70	0.144	0.023	0.10
	ON rF3-2	23.00	3.06		0.38	0.77				
	ON rF3-3	23.06	3.12		0.44	0.74				
β-actin	OFF 1	19.51								
	OFF 2	19.64								
	OFF 3	20.31								
	ON rGFP-1	19.05								
	ON rGFP-2	18.43								
	ON rGFP-3	20.10								
	ON rF3-1	20.02								
	ON rF3-2	19.94								
	ON rF3-3	19.94								

Target Name	Sample Name	Ct(SDM)	ΔCt	ave Δct	ΔΔct	2^(-ΔΔCt)	ave fold change	v.s. - t test	v.s. rGFP t test	sd
CDH3 (P-cad)	OFF 4	24.91	8.67	8.66	0.01	1.00	1.00			0.02
	OFF 5	25.78	8.63		-0.03	1.02				
	OFF 6	25.13	8.69		0.03	0.98				
	ON rGFP-4	21.04	4.12		-4.54	23.32	16.29	0.027		6.47
	ON rGFP-5	21.40	4.76		-3.90	14.96				
	ON rGFP-6	22.07	5.26		-3.40	10.58				
	ON rF3-4	23.79	6.87		-1.79	3.47	5.75	0.042	0.076	2.57
	ON rF3-5	22.24	5.57		-3.09	8.53				
	ON rF3-6	23.03	6.27		-2.39	5.25				
T (brachyury)	OFF 4	25.17	8.93	8.92	0.01	0.99	1.00			0.01
	OFF 5	26.07	8.92		0.00	1.00				
	OFF 6	25.34	8.90		-0.02	1.01				
	ON rGFP-4	22.24	5.32		-3.60	12.10	8.00	0.042		3.75
	ON rGFP-5	22.72	6.08		-2.84	7.14				
	ON rGFP-6	23.48	6.67		-2.25	4.75				
	ON rF3-4	23.25	6.33		-2.59	6.01	3.69	0.074	0.032	2.01
	ON rF3-5	24.28	7.61		-1.31	2.47				
	ON rF3-6	24.31	7.55		-1.37	2.58				
β-actin	OFF 4	16.24								
	OFF 5	17.15								
	OFF 6	16.44								
	ON rGFP-4	16.92								
	ON rGFP-5	16.64								
	ON rGFP-6	16.81								
	ON rF3-4	16.92								
	ON rF3-5	16.67								
	ON rF3-6	16.76								

Analysis of each experimental group

Target Name	Sample Name	ΔCt	ΔΔCt	2^(-ΔΔCt)
Nanog	OFF1	3.48	0	1
	ON rGFP-1	5.18	1.7	0.31
	ON rF3-1	3.46	-0.02	1.01

Target Name	Sample Name	ΔCt	ΔΔCt	2^(-ΔΔCt)
CDH3	OFF4	8.67	0	1
	ON rGFP-4	4.12	-4.55	23.43
	ON rF3-4	6.87	-1.8	3.48

Target Name	Sample Name	ΔCt	ΔΔCt	2^(-ΔΔCt)
T	OFF4	8.93	0	1
	ON rGFP-4	5.32	-3.61	12.21
	ON rF3-4	6.33	-2.6	6.06

Nanog	OFF2	2.62	0	1
	ON rGFP-2	4.27	1.65	0.32
	ON rF3-2	3.06	0.44	0.74

CDH3	OFF5	8.63	0	1
	ON rGFP-5	4.76	-3.87	14.62
	ON rF3-5	5.57	-3.06	8.34

T	OFF5	8.92	0	1
	ON rGFP-5	6.08	-2.84	7.16
	ON rF3-5	7.61	-1.31	2.47

Nanog	OFF3	1.94	0	1
	ON rGFP-3	3.56	1.62	0.33
	ON rF3-3	3.12	1.18	0.44

CDH3	OFF6	8.69	0	1
	ON rGFP-6	5.26	-3.43	10.78
	ON rF3-6	6.27	-2.42	5.35

T	OFF6	8.9	0	1
	ON rGFP-6	6.67	-2.23	4.69
	ON rF3-6	7.55	-1.35	2.55

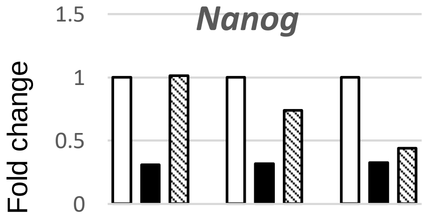

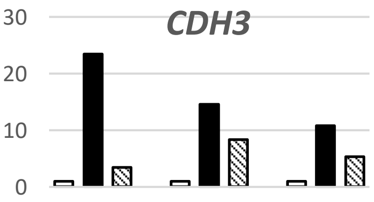

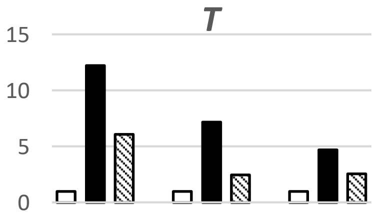

Raw data and statistical analysis for Fig. 2D

Fig. 2D								v.s. -day0	v.s. -	
Target Name	Sample Name	Ct(SDM)	ΔCt	ave Δct	ΔΔct	2^(-ΔΔCt)	ave fold change	t test	t test	sd
	- day0	25.36	8.43		0.57	0.68				
	- day0	24.32	7.33	7.86	-0.53	1.45	1.05			0.39
	- day0	24.73	7.83		-0.03	1.02				
	-	26.49	9.36		1.50	0.35				
	-	26.98	10.32		2.46	0.18	0.24	0.025		0.10
	-	26.93	10.21		2.35	0.20				
	cont pep.	26.21	10.01		2.15	0.23				
	cont pep.	26.06	9.23		1.37	0.39	0.25	0.027	0.912	0.12
	cont pep.	26.71	10.59		2.73	0.15				
	rF3	25.02	7.74		-0.12	1.09				
Nanog	rF3	25.61	7.24		-0.62	1.54	1.10	0.886	0.029	0.44
	rF3	25.30	8.44		0.58	0.67				
	St4n1	24.71	8.39		0.53	0.69				
	St4n1	24.09	7.20		-0.66	1.58	1.08	0.937	0.037	0.46
	St4n1	24.49	7.93		0.07	0.95				
	FAK act.	24.66	8.31		0.45	0.73				
	FAK act.	24.49	8.11		0.25	0.84	1.07	0.952	0.047	0.49
	FAK act.	23.99	7.15		-0.71	1.64				
	Rho/ROCK inh.	26.81	10.16		2.30	0.20				
	Rho/ROCK inh.	26.64	9.65		1.79	0.29	0.30	0.016	0.528	0.10
	Rho/ROCK inh.	26.21	9.16		1.30	0.41				
	- day0	31.69	14.76		1.32	0.40				
	- day0	30.13	13.14	13.44	-0.30	1.23	1.22			0.81
	- day0	29.33	12.43		-1.01	2.02				
	-	27.26	10.13		-3.31	9.94				
	-	28.88	12.22		-1.22	2.33	6.31	0.043		3.81
	-	27.43	10.71		-2.73	6.65				
	cont pep.	27.18	10.98		-2.46	5.51				
	cont pep.	26.04	9.21		-4.23	18.81	10.93	0.037	0.371	6.98
	cont pep.	26.48	10.36		-3.08	8.48				
	rF3	29.56	12.28		-1.16	2.24				
T (brachyury)	rF3	32.35	13.98		0.54	0.69	1.15	0.933	0.043	0.94
	rF3	31.21	14.35		0.91	0.53				
	St4n1	29.40	13.08		-0.36	1.29				
	St4n1	29.44	12.55		-0.89	1.86	1.41	0.737	0.046	0.41
	St4n1	29.90	13.34		-0.10	1.07				
	FAK act.	29.91	13.56		0.12	0.92				
	FAK act.	29.64	13.26		-0.18	1.14	1.20	0.978	0.041	0.32
	FAK act.	29.65	12.81		-0.63	1.55				
	Rho/ROCK inh.	28.77	12.12		-1.32	2.50				
	Rho/ROCK inh.	27.74	10.75		-2.69	6.47	5.33	0.026	0.729	2.47
	Rho/ROCK inh.	27.68	10.63		-2.81	7.03				
	- day0	23.43	6.50		0.19	0.87				
	- day0	24.11	7.12	6.31	0.81	0.57	1.15			0.76
	- day0	22.20	5.30		-1.01	2.01				
	-	20.39	3.26		-3.05	8.26				
	-	20.46	3.80		-2.51	5.68	6.82	0.001		1.32
	-	20.32	3.60		-2.71	6.53				
	cont pep.	21.59	5.39		-0.92	1.89				
	cont pep.	20.95	4.12		-2.19	4.55	2.98	0.118	0.013	1.40
	cont pep.	21.11	4.99		-1.32	2.49				
	rF3	22.33	5.61		-0.70	1.62				
CDH3 (P-cad)	rF3	23.21	5.93		-0.38	1.30	1.28	0.800	0.002	0.35
	rF3	24.79	6.42		0.11	0.92				
	St4n1	22.57	5.52		-0.79	1.73				
	St4n1	23.89	7.57		1.26	0.42	1.17	0.974	0.003	0.68
	St4n1	22.74	5.85		-0.46	1.37				
	FAK act.	22.05	5.49		-0.82	1.76				
	FAK act.	21.91	5.56		-0.75	1.68	1.35	0.742	0.003	0.64
	FAK act.	23.38	7.00		0.69	0.62				
	Rho/ROCK inh.	20.29	3.43		-2.88	7.34				
	Rho/ROCK inh.	20.92	4.27		-2.04	4.10	5.27	0.011	0.294	1.80
	Rho/ROCK inh.	21.17	4.18		-2.13	4.37				
	- day0	16.93								
	- day0	16.99								
	- day0	16.90								
	-	17.13								
	-	16.66								
	-	16.72								
	cont pep.	16.20								
	cont pep.	16.83								
	cont pep.	16.12								
	rF3	17.28								
β-actin	rF3	18.37								
	rF3	16.86								
	St4n1	16.32								
	St4n1	16.89								
	St4n1	16.56								
	FAK act.	16.35								
	FAK act.	16.38								
	FAK act.	16.84								
	Rho/ROCK inh.	16.65								
	Rho/ROCK inh.	16.99								
	Rho/ROCK inh.	17.05								

Raw data and statistical analysis for Fig. 3B

Fig3B								vs OFF	vs -	
Target Name	Sample Name	Ct(SDM)	ΔCt	ave Δct	ΔΔct	2^(-ΔΔCt)	ave fold change	t test	t test	sd
Nanog	OFF	17.92	2.25	2.40	-0.15	1.11	1.00			0.09
	OFF	18.32	2.46		0.06	0.96				
	OFF	18.08	2.48		0.08	0.94				
	ON -	19.22	3.23		0.83	0.56	0.54	0.001		0.03
	ON -	19.26	3.28		0.88	0.54				
	ON -	18.99	3.39		0.99	0.50				
	ON FAK act.	18.80	3.40		1.00	0.50	0.49	0.001	0.063	0.01
	ON FAK act.	18.89	3.47		1.07	0.48				
	ON FAK act.	18.89	3.43		1.03	0.49				
T (brachyury)	OFF	28.22	12.55	12.61	-0.06	1.04	1.00			0.08
	OFF	28.39	12.53		-0.08	1.06				
	OFF	28.35	12.75		0.14	0.91				
	ON -	27.01	11.02		-1.59	3.01	2.77	0.0004		0.27
	ON -	27.28	11.30		-1.31	2.48				
	ON -	26.72	11.12		-1.49	2.81				
	ON FAK act.	27.99	12.59		-0.02	1.01	1.13	0.161	0.001	0.10
	ON FAK act.	27.78	12.36		-0.25	1.19				
	ON FAK act.	27.81	12.35		-0.26	1.20				
CDH3 (p-cad)	OFF	26.88	11.21	10.85	0.36	0.78	1.02			0.25
	OFF	26.72	10.86		0.01	1.00				
	OFF	26.09	10.49		-0.36	1.29				
	ON -	24.96	8.97		-1.88	3.69	3.65	0.001		0.40
	ON -	24.82	8.84		-2.01	4.04				
	ON -	24.76	9.16		-1.69	3.23				
	ON FAK act.	26.54	11.14		0.29	0.82	0.72	0.139	0.000	0.12
	ON FAK act.	26.67	11.25		0.40	0.76				
	ON FAK act.	27.08	11.62		0.77	0.59				
β-actin	OFF	15.67								
	OFF	15.86								
	OFF	15.60								
	ON -	15.99								
	ON -	15.98								
	ON -	15.60								
	ON FAK act.	15.40								
	ON FAK act.	15.42								
	ON FAK act.	15.46								

Raw data and statistical analysis for Fig. 3C

Fig 3C									
Target Name	Sample Name	Ct(SDM)	ΔCt	ave Δct	ΔΔct	2^(-ΔΔCt)	ave fold change	t test	sd
Nanog	-	19.00	2.33	2.20	0.13	0.92	1.00		0.10
	-	18.22	2.05		-0.15	1.11			
	-	18.23	2.23		0.03	0.98			
	FAK inh.	18.82	2.16		-0.04	1.03	0.91	0.616	0.28
	FAK inh.	18.99	2.05		-0.15	1.11			
	FAK inh.	19.00	2.98		0.78	0.58			
T (brachyury)	-	25.46	8.79	9.02	-0.23	1.17	1.02		0.24
	-	24.98	8.81		-0.21	1.15			
	-	25.45	9.45		0.43	0.74			
	FAK inh.	24.07	7.41		-1.61	3.05	2.44	0.025	0.66
	FAK inh.	24.62	7.68		-1.34	2.53			
	FAK inh.	24.24	8.22		-0.80	1.74			
CDH3 (P-cad)	-	27.05	10.38	11.32	-0.94	1.92	1.12		0.69
	-	27.78	11.61		0.29	0.82			
	-	27.97	11.97		0.65	0.64			
	FAK inh.	27.42	10.76		-0.56	1.47	1.21	0.862	0.48
	FAK inh.	27.67	10.73		-0.59	1.51			
	FAK inh.	27.93	11.91		0.59	0.66			
β-actin	-	16.67							
	-	16.17							
	-	16.00							
	FAK inh.	16.66							
	FAK inh.	16.94							
	FAK inh.	16.02							

Raw data and statistical analysis for Fig. 3D

Fig 3D									
Target Name	Sample Name	Ct(SDM)	ΔCt	ave Δct	ΔΔct	2^(-ΔΔCt)	ave fold change	t test	sd
Nanog	-	19.72	3.49	3.55	-0.06	1.04	1.01		0.13
	-	19.76	3.76		0.21	0.86			
	-	19.63	3.39		-0.16	1.11			
	FAK act.	20.98	4.32		0.77	0.59	0.75	0.092	0.16
	FAK act.	20.52	3.70		0.15	0.90			
	FAK act.	20.09	3.95		0.40	0.76			
T (brachyury)	-	25.00	8.77	8.94	-0.17	1.12	1.00		0.10
	-	25.05	9.05		0.11	0.92			
	-	25.23	8.99		0.05	0.96			
	FAK act.	26.72	10.06		1.12	0.46	0.34	0.002	0.11
	FAK act.	27.69	10.87		1.93	0.26			
	FAK act.	26.88	10.74		1.80	0.29			
CDH3 (P-cad)	-	25.58	9.35	9.51	-0.16	1.11	1.01		0.21
	-	25.43	9.43		-0.08	1.05			
	-	25.98	9.74		0.23	0.85			
	FAK act.	25.83	9.17		-0.34	1.26	1.27	0.251	0.37
	FAK act.	25.65	8.83		-0.68	1.60			
	FAK act.	25.70	9.56		0.05	0.96			
β-actin	-	16.23							
	-	16.00							
	-	16.24							
	FAK act.	16.66							
	FAK act.	16.82							
	FAK act.	16.14							

Raw data and statistical analysis for Fig. 4C

Fig 4C									
Target Name	Sample Name	Ct(SDM)	ΔCt	ave Δct	ΔΔct	2^(-ΔΔCt)	ave fold change	t test	sd
Nanog	-	24.35	7.92	7.82	0.10	0.93	1.01		0.21
	-	24.54	7.49		-0.33	1.25			
	-	25.34	8.04		0.22	0.86			
	PI3K inh.	25.35	9.14		1.32	0.40	0.47	0.015	0.10
	PI3K inh.	25.93	9.04		1.22	0.43			
	PI3K inh.	25.55	8.60		0.78	0.58			
T (brachyury)	-	30.56	14.13	14.13	0.00	1.00	1.00		0.00
	-	31.18	14.13		0.00	1.00			
	-	31.42	14.12		-0.01	1.00			
	PI3K inh.	28.64	12.43		-1.70	3.24	4.72	0.018	1.67
	PI3K inh.	28.88	11.99		-2.14	4.40			
	PI3K inh.	28.37	11.42		-2.71	6.53			
CDH3 (P-cad)	-	27.81	11.38	10.61	0.77	0.59	1.07		0.43
	-	27.16	10.11		-0.50	1.42			
	-	27.65	10.35		-0.26	1.20			
	PI3K inh.	23.61	7.40		-3.21	9.27	7.56	0.003	1.72
	PI3K inh.	24.96	8.07		-2.54	5.83			
	PI3K inh.	24.64	7.69		-2.92	7.59			
β-actin	-	16.43							
	-	17.05							
	-	17.30							
	PI3K inh.	16.21							
	PI3K inh.	16.89							
	PI3K inh.	16.95							

Raw data and statistical analysis for Fig. 6B

Fig 6B								vs OFF	vs -	
Target Name	Sample Name	Ct(SDM)	ΔCt	ave Δct	ΔΔct	2^(-ΔΔCt)	ave fold change	t test	t test	sd
Nanog	OFF	22.77	2.87	3.00	-0.13	1.10	1.00			0.11
	OFF	22.73	2.94		-0.06	1.04				
	OFF	22.19	3.19		0.19	0.88				
	ON -	24.04	4.13		1.13	0.46	0.37	0.001		0.08
	ON -	24.21	4.45		1.45	0.37				
	ON -	24.15	4.76		1.76	0.30				
	ON Rho/ROCK inh.	24.67	5.23		2.23	0.21	0.24	0.014	0.214	0.05
	ON Rho/ROCK inh.	24.61	5.17		2.17	0.22				
	ON Rho/ROCK inh.	24.17	4.75		1.75	0.30				
T (brachyury)	OFF	30.88	10.98	11.13	-0.15	1.11	1.04			0.33
	OFF	30.51	10.72		-0.40	1.32				
	OFF	30.69	11.68		0.55	0.68				
	ON -	27.19	7.28		-3.85	14.45	10.33	0.045		3.57
	ON -	27.85	8.09		-3.04	8.23				
	ON -	27.47	8.07		-3.06	8.32				
	ON Rho/ROCK inh.	27.29	7.86		-3.27	9.65	9.99	0.002	0.891	0.34
	ON Rho/ROCK inh.	27.25	7.81		-3.32	9.98				
	ON Rho/ROCK inh.	27.18	7.76		-3.37	10.33				
CDH3 (P-cad)	OFF	30.83	10.93	11.05	-0.12	1.08	1.01			0.16
	OFF	30.68	10.89		-0.16	1.12				
	OFF	30.33	11.33		0.28	0.83				
	ON -	28.18	8.27		-2.78	6.87	6.10	0.007		0.89
	ON -	28.15	8.39		-2.66	6.32				
	ON -	28.08	8.69		-2.36	5.13				
	ON Rho/ROCK inh.	28.02	8.58		-2.47	5.53	5.48	0.00003	0.303	0.12
	ON Rho/ROCK inh.	28.01	8.57		-2.48	5.57				
	ON Rho/ROCK inh.	28.05	8.63		-2.42	5.35				
β-actin	OFF	19.90								
	OFF	19.79								
	OFF	19.00								
	ON -	19.91								
	ON -	19.76								
	ON -	19.39								
	ON Rho/ROCK inh.	19.44								
	ON Rho/ROCK inh.	19.44								
	ON Rho/ROCK inh.	19.42								

Nozaki S et al., Supplementary Fig S4

mESCs

Without 2i and LIF

Target Name	Sample Name	Ct(SDM)	ΔCt	ave Δct	ΔΔct	fold change	ave fold change	t test	sd
T (brachyury)	-	24.86	8.19		-0.23	1.17			
	-	24.68	8.51	8.42	0.09	0.94	1.01		0.14
	-	24.55	8.55		0.13	0.91			
	FAK act.	26.83	10.17		1.75	0.30			
	FAK act.	26.76	9.82		1.40	0.38	0.35	0.002	0.04
	FAK act.	25.88	9.86		1.44	0.37			
β-actin	-	16.67							
	-	16.17							
	-	16.00							
	FAK act.	16.66							
	FAK act.	16.94							
	FAK act.	16.02							
Target Name	Sample Name	Ct(SDM)	ΔCt	ave Δct	ΔΔct	fold change	ave fold change	t test	sd
CDH3 (P-cad)	-	24.45	7.58		-0.07	1.05			
	-	24.20	7.21	7.65	-0.44	1.36	1.04		0.33
	-	24.98	8.16		0.51	0.70			
	FAK act.	24.91	8.91		1.26	0.42			
	FAK act.	24.63	8.31		0.66	0.63	0.49	0.053	0.13
	FAK act.	24.93	8.95		1.30	0.41			
β-actin	-	16.87							
	-	16.99							
	-	16.82							
	FAK act.	16.00							
	FAK act.	16.32							
	FAK act.	15.98							

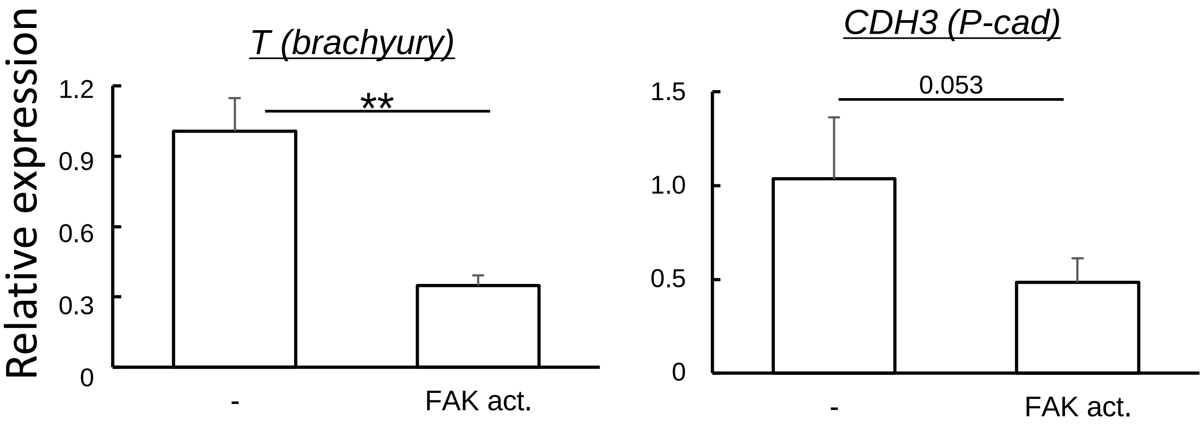

The activation of FAK protects against the upregulation of gastrulation markers in mESCs cultured without stemness factors 2i and LIF. Upon removal of 2i and LIF, the gastrulation markers *T* (*brachyury*) and the gastrulation onset marker *CDH3* (*P-cadherin*) began to express within two days, a process significantly attenuated by the FAK activator ZINC 40099027. n=3, **; p< 0.01
